# Editorial: Lipids, lipid oxidation, and cancer: from biology to therapeutics

**DOI:** 10.3389/fonc.2024.1414992

**Published:** 2024-04-19

**Authors:** Sergio P. Bydlowski, Marc Poirot

**Affiliations:** ^1^ Lipids, Oxidation and Cell Biology Team, Laboratory of Immunology (LIM19), Heart Institute (InCor), Faculdade de Medicina, Universidade de São Paulo, Sao Paulo, Brazil; ^2^ Department of Hematology, Hemotherapy and Cell Therapy, Faculdade de Medicina, Universidade de São Paulo, Sao Paulo, Brazil; ^3^ National Institute of Science and Technology in Regenerative Medicine (INCT-Regenera), CNPq, Rio de Janeiro, Brazil; ^4^ Cancer Research Center of Toulouse (CRCT), Inserm, CNRS, University of Toulouse III, Team INOV: “Cholesterol Metabolism and Therapeutic Innovations”, Toulouse, France; ^5^ French Network for Nutrition Physical Activity and Cancer Research (NACRe Network), Jouy-en-Josas, France

**Keywords:** lipids, lipid oxidation, lipoprotein, cancer, immune cells, fatty acid, triple-negative breast cancer, HPV

From fatty acids, the most fundamental biological lipid, glycerolipids, phospholipids, and sphingolipids, to lipoproteins and steroid molecules such as cholesterol, oxysterols, vitamin D, lipids all have distinct and multiple biological activities and functions. Several lipid metabolic aspects such as biosynthesis, oxidation, uptake, enzymes, regulation, signaling pathways, have been shown to be implicated in several diseases, including cancer.

In fact, lipids are crucial in the pathophysiology of cancer development. Several types of cancers share common alterations in the complex cell lipid metabolism. These dysregulated changes can affect several physiological characteristics of cells such as membrane synthesis, energy homeostasis, post-translational protein modifications, and cell signaling, thus sustaining cell growth, proliferation, differentiation, and survival (1–6), the most prominent features of cancer cells, as is evident by the articles in this topic.

The work of Duong et al. provides a review of the state of the art in lipid metabolism in tumor immunology. Lipids are an important source of energy for rapidly proliferating cells. Lipids also affect the immune system and its components in a variety of ways. Accumulation of lipids in the tumor microenvironment has been shown to promote immune evasion and inflammation In fact, abnormal lipid accumulation in tumors correlates with T-cell dysfunction, T-cell exhaustion, increased proportions of regulatory T cells and memory T cells, and increased T-cell recall responses.

The authors addressed the importance of lipid metabolism by describing the action of lipids and lipid oxidation in 1. Immune cells (T cells, macrophages, natural killer cells, dendritic cells); 2. The proliferation and survival of cancer cells; 3. Cancer progression and angiogenesis (overview of lipid metabolism, role of lipid metabolism in cancer); 4. Cancer metastasis; and 5. Cancer immunotherapy (lipids as adjuvants, lipids as vehicles, role of lipids in immune responsiveness.

Triple-negative breast cancer (TNBC) accounts for 10-20% of all breast cancer. It is negative for hormone receptors (estrogen, progesterone) and HER-2. Changes in plasma lipid and lipoprotein profiles contributes to BC, considering the role of lipids, particularly cholesterol, in tumor proliferation and metastasis (7, 8). Campos et al. describes that HDL retards LDL oxidation by 22% in the plasma of TNBC patients as compared to the control group and this is positively correlated with apoA-I content in HDL. Moreover, the antioxidant activity of HDL was greater in the advanced stages of TNBC. The findings highlight the role of HDL as an antioxidant defense in limiting oxidative and inflammatory stress in advanced stages of TNBC.

The next two articles are related to the effects of Human Papilloma Virus (HPV). The main cause of preinvasive or invasive cervical cancer is infection with HPV. Loss of apoptotic control allows cancer cells to survive longer, increasing both, the time for mutation accumulation and the ability to invade during tumor development. After infection, HPV encodes proteins E6 and E7 which together promote cell proliferation, prolong cell cycle progression, and prevent apoptosis (9, 10). E6 and E7 initiate oncogenesis through interactions with tumor suppressor genes -TP53 for E6 and retinoblastoma protein for E7 (11).

Liu et al. findings are related to the metabolic heterogeneity in cervical cancer cell lines C33A and CaSki, evaluated by multiomics analysis. The differential metabolites were screened, and functional enrichment and pathway analysis were performed. Association analysis was carried out with transcriptomics, and the important differential metabolisms were analyzed by real-time PCR. The findings showed differences in amino acids, nucleotides and lipids (such as threonine, arachidonic acid and hypoxanthine) in the metabolic pathways between the C33A and CaSki cell lines. C33A cells exhibited higher contents of fatty acid polar derivatives, while CaSki cells showed higher contents of free fatty acids and glycerides. The findings suggest that p53 and the genes involved in lipid metabolism pathways, such as PPARG and SCD, are relevant to the metabolic heterogeneity of the cells. In summary, their results showed that the metabolomic differences between C33A and CaSki cells might be related to the decreased expression of PPARG and p53 caused by HPV E6.

The work by Permatasari et al. aimed to evaluate the anticancer activity of *Caulerpa racemosa* on HeLa cervical cancer cells. Natural sources, especially underutilized marine products, have promising potential as functional food or nutraceutical with anticancer properties. In fact, natural marine products, as sea grapes, have been used as compounds for drug discovery. They are frequently used as drugs (antiageing, antidiabetic, antirheumatism). Sea grapes is a term for varieties of green seaweed of the genus Caulerpa (12). Caulerpa has bioactive metabolites (alkaloids, terpenoids, flavonoids, steroids and tannins) and its bioactivity has been reported against cancer (13). This article describes that *C. racemosa* extract significantly increases the expression of pro-apoptotic proteins BAX and cleaves caspase-3. Annexin V-PI induced apoptosis in treated cells and decreased HeLa cell viability at 24 h and 48 h post-treatment. There has been no research up to now on the benefits of *C. racemosa* originating from Indonesia. The potential of this *C. racemosa* as an anticancer by inhibiting antiapoptosis (Bcl-2), increasing proapoptosis (BAX) and cleaved caspase-3 *in vitro* is here described for the first time.

In the opinion article discussing the combination of green seaweed’s fatty acids and heterocyclic derivatives as anticancer nutraceuticals by Taher et al., they discuss the future implication and direction of this application. Green seaweed has fatty acid content that makes up most of its fat content. The incorporation of heterocyclic compounds into fatty acids are proposed to increase the anticancer cytotoxicity and efficacy of cancer therapeutic agents. Therefore, green seaweeds would have their potential as anticancer nutraceuticals.

Finally, in the last article (14), Nurkolis et al. proposed the soy-based tempe as part of a future meal by its anti cancer potential. In fact, a diet high in soy has been associated with a lower prevalence of several types of cancer (14). Tempe, a soy-based fermented food originating from Indonesia, is reported to be capable of inhibiting proliferation and angiogenesis as well as triggering apoptosis in cancer cells (15). This opinion paper presents updated evidence about the anticancer potential of soy-based tempe and the possibility of its use as a functional meal.

In conclusion, the articles that comprise this Research Topic highlight several key roles that lipids can play in several aspects of cancer and address ongoing and future challenges.

## Author contributions

SB: Conceptualization, Formal analysis, Supervision, Writing – original draft, Writing – review & editing. MP: Conceptualization, Formal analysis, Supervision, Writing – original draft, Writing – review & editing.
